# Ion channels in the central regulation of energy and glucose homeostasis

**DOI:** 10.3389/fnins.2013.00085

**Published:** 2013-05-23

**Authors:** Jong-Woo Sohn

**Affiliations:** Division of Hypothalamic Research, Department of Internal Medicine, The University of Texas Southwestern Medical CenterDallas, TX, USA

**Keywords:** patch clamp electrophysiology, obesity, diabetes mellitus, K^+^ channels, TRPC channels, ionotropic glutamate receptors (iGluRs), GABA_A_ receptors

## Abstract

Ion channels are critical regulators of neuronal excitability and synaptic function in the brain. Recent evidence suggests that ion channels expressed by neurons within the brain are responsible for regulating energy and glucose homeostasis. In addition, the central effects of neurotransmitters and hormones are at least in part achieved by modifications of ion channel activity. This review focuses on ion channels and their neuronal functions followed by a discussion of the identified roles for specific ion channels in the central pathways regulating food intake, energy expenditure, and glucose balance.

## Introduction

Energy and glucose homeostasis is tightly regulated by mechanisms within the central nervous system (CNS) (Williams and Elmquist, 2012). Central neurons integrate peripheral and central signals for a coordinated modulation of food intake, energy expenditure, and glucose homeostasis (Williams and Elmquist, 2012). In recent years, researchers have utilized the Cre-loxP technology to selectively delete or reactivate receptors and signaling molecules of interest. Although this genetic strategy has some limitations including germ line expression of Cre transgenes and developmental compensation of missing molecules (Padilla et al., 2010, 2012; Morrison and Munzberg, 2012), the Cre-loxP technology has greatly contributed to determine the *in vivo* physiological roles for receptors, signaling molecules, neuropeptides and neurotransmitters in a neuron-specific manner. However, relatively little is known about the *in vivo* physiological function of ion channels expressed by central neurons. Importantly, neurotransmitters and hormones frequently alter the activity of ion channels to modify neuronal function involved in the central regulation of metabolism. Combined with mouse genetics and neuroanatomical approaches, electrophysiological techniques have been successfully applied to uncover novel roles for ion channels in the regulation of neurons by neurotransmitters and hormones (Cowley et al., 2001; Qiu et al., 2010; Klockener et al., 2011; Sohn et al., 2011, 2013; Vong et al., 2011; Cui et al., 2012; Liu et al., 2012). This review will focus on several ion channels that have been found to regulate neuronal function *in vitro* and/or to have metabolic effects *in vivo*. Thus, recent advances and current challenges in understanding the role of ion channels in central neurons regulating metabolism will be discussed.

## Role of ion channels in neuronal activity

**Figure 1:**
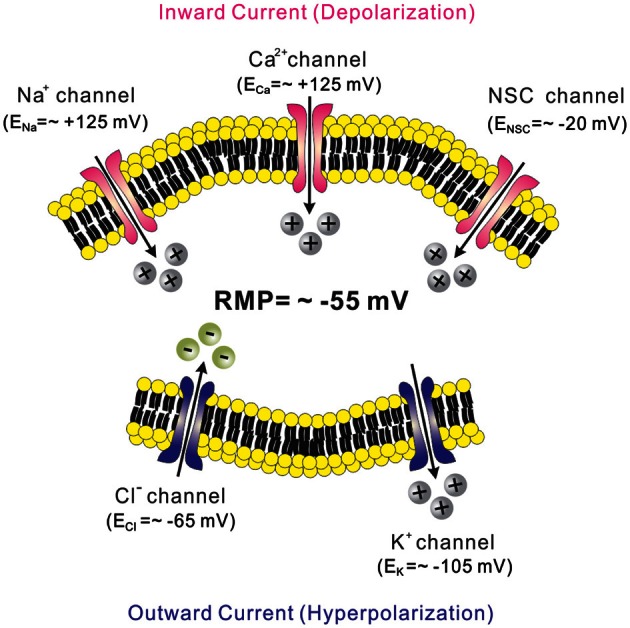
**Ion channels and regulation of membrane excitability.** Neurons within the arcuate nucleus typically have a RMP of ~55 mV (*See text*). Cations rush in through open channels when RMP is more negative to the equilibrium potentials (pink-colored channels). By contrast, cations rush out or anions rush in through open channels when RMP is more positive to the equilibrium potentials (blue-colored channels). Equilibrium potentials were calculated using Nernst equation assuming experimental conditions in Sohn et al. (2011).

Ion channels are critical in regulating the membrane potential of neurons (Hille, 2001). Typically, the activation of a specific ion channel will either activate or inhibit a neuron depending on the resting membrane potential (RMP) and the ion's equilibrium potential (Figure 1). If RMP is more negative than the equilibrium potential of an ion, the cation will rush in through open channels (inward current) in the cell membrane which results in a depolarization of the membrane potential (e.g., Na^+^ channels, Ca^2+^ channels, and non-selective cation or NSC channels, Figure 1, pink-colored channels). By contrast, cations rush out or anions rush in through open channels (outward current) to hyperpolarize membrane potential when RMP is more positive than the equilibrium potential of an ion (e.g., K^+^ channels and Cl^−^ channels, Figure 1, blue-colored channels). Typically, neurons within the arcuate nucleus of hypothalamus have a RMP of ~55 mV (Cowley et al., 2001; Hill et al., 2008; Al-Qassab et al., 2009; Williams et al., 2010; Sohn et al., 2011) (Figure 1). Thus, the activation of NSC channels will depolarize a cell because the estimated E_NSC_ is approximately −20 mV (Cowley et al., 2001; Hill et al., 2008; Williams et al., 2010; Sohn et al., 2011) (Figure 1). By contrast, activation of K^+^ channels will hyperpolarize the membrane potential because E_K_ is approximately −105 mV (Williams et al., 2010; Sohn et al., 2013) (Figure 1).

The direct activation of most ion channels is triggered by either changes in voltage (voltage-gated channels) or neurotransmitters and hormones (ligand-gated channels). For instance, direct depolarization of neurons by electrical stimuli will open voltage-gated Na^+^, Ca^2+^, and K^+^ channels, which contribute to action potentials. Examples of ligand-gated channels include ionotropic receptors of glutamate and gamma-aminobutyric acid (GABA). While the slow actions of these neurotransmitters are mediated by the G-protein coupled metabotropic receptors (mGluRs and GABA_B_ receptors), their fast actions are mediated by the ionotropic receptors (iGluRs and GABA_A_ receptors) (Hammond, 2001). Glutamate released from presynaptic terminals activates the iGluRs (AMPA, kainate, or NMDA receptors; these are NSC channels) to generate excitatory postsynaptic potentials (EPSPs) (Hammond, 2001). Likewise, GABA activates the ionotropic GABA_A_ receptors (these are chloride channels) to generate inhibitory postsynaptic potentials (IPSPs) (Hammond, 2001). In addition to these direct modes of ion channel activation, neurotransmitters and hormones bind to their cognate receptors and activate cellular signaling cascades, which ultimately results in the regulation of ion channel activity.

## Ion channels in the central regulation of metabolism

### Potassium channels

Given the large contribution of the K^+^ conductance to RMP, neurotransmitters and hormones frequently target K^+^ channels to modify cellular activity. Once activated, K^+^ channels hyperpolarize the membrane potential and inhibit neuronal activity. If K^+^ channels are active at rest and contribute to stabilizing the membrane potential, the inhibition of K^+^ channels depolarizes membrane potential and enhances neuronal activity. As discussed below, two inward rectifier K^+^ channels (ATP-sensitive K^+^ channels and G protein-gated inwardly rectifying K^+^ channels) have been found to mediate central regulation of food intake, energy expenditure and glucose homeostasis.

#### ATP-sensitive K^+^ (K_ATP_) channels

K_ATP_ (Kir6) channels belong to a subfamily of inward rectifier K^+^ channels gated by changes in intracellular ATP levels (Hibino et al., 2010). Functional K_ATP_ channels consist of four pore forming subunits Kir6.x (Kir6.1 or Kir6.2) and four regulatory subunits SURx (SUR1, SUR2A, or SUR2B) (Flagg et al., 2010; Hibino et al., 2010). Typically, K_ATP_ channel compositions are Kir6.2 + SUR1 in pancreatic beta cells, Kir6.2 + SUR2A in cardiac muscle cells, Kir6.1 + SUR2B in smooth muscle cells (Hibino et al., 2010). Low ATP levels open K_ATP_ channels while high ATP levels close these channels. For instance, in hyperglycemic conditions with elevated intracellular ATP levels, the inhibition of K_ATP_ channels leads to depolarization of pancreatic beta cells and insulin secretion (Hibino et al., 2010). By contrast, low intracellular ATP levels such as in coronary ischemia results in the opening of cardiac K_ATP_ channels and a hyperpolarization, which stabilizes cardiac myocytes (Flagg et al., 2010; Hibino et al., 2010). This is thought to protect the heart from further injury. Thus, K_ATP_ channels transform cellular energy status to electrical activity in pathophysiological settings.

***K_ATP_ and other channels sense glucose levels in brain***. K_ATP_ channels are widely expressed throughout the brain (Ashcroft, 1988). It is believed that neurons expressing K_ATP_ channels sense brain glucose levels and regulate glucose homeostasis by changing their excitability (Ashcroft, 1988). Elevated brain glucose levels activate glucose-excited (or glucose-reponsive) neurons via the inhibition of K_ATP_ channels in multiple brain areas including the ventromedial hypothalamic nucleus (VMH), the arcuate nucleus, and the lateral hypothalamic area (LHA) (Ashford et al., 1990; Song et al., 2001; Routh, 2002; Ibrahim et al., 2003; Wang et al., 2004; Burdakov et al., 2005; Claret et al., 2007) (Table 1). In brain, Kir6.2 has been suggested to be the pore-forming subunits (Karschin et al., 1998; Zawar et al., 1999; Miki et al., 2001), and defective Kir6.2 subunits resulted in non-functional K_ATP_ channels. For instance, it was demonstrated that genetic deletion of Kir6.2 subunits deprived VMH neurons of K_ATP_ currents as well as glucose responsiveness, and that glucagon secretion was defective in these mice (Miki et al., 2001). Notably, pancreatic alpha cell activity was normal in Kir6.2 knockout mice (Miki et al., 2001). It was also demonstrated that defective Kir6.2 subunits in specific neurons affect glucose homeostasis. For instance, mutant Kir6.2 subunits in arcuate pro-opiomelanocortin (POMC) neurons led to impaired POMC neuron glucose responsiveness and impaired whole body glucose tolerance (Parton et al., 2007). Moreover, melanin-concentrating hormone (MCH) neurons within LHA were not activated by elevated glucose levels when Kir6.2 subunits in these neurons lacked glucose-sensing amino acid residues: these mice also showed impaired glucose tolerance (Kong et al., 2010). Therefore, Kir6.2 subunits expressed by VMH neurons, arcuate POMC neurons, and LHA MCH neurons mediate glucose-excitation of these neurons. In addition, these results suggest that glucose sensing by these neurons are required for regulating whole body glucose homeostasis.

**Table 1:** **Acute cellular effects of metabolic signals in the CNS**.

**Metabolic signal**	**Inhibited neurons**	**Activated neurons**
Elevated glucose	Orexin/Hypocretin neuron (↑K2P channel)	VMH neuron (↓K_ATP_ channel)
	Arcuate nucleus neuron (↑CFTR)	POMC neuron (↓K_ATP_ channel)
	VMH neuron (↑CFTR)	MCH neuron (↓K_ATP_ channel)
Leptin	NPY/AgRP neuron (↑K_ATP_ channel)	POMC neuron (↑TRPC channel)
	SF1 neuron (^*^N.D.)	Kiss1 neuron (↑TRPC channel)
	PMV neuron (↑K_ATP_ channel)	SF1 neuron (N.D.)
	LHA MC4R neuron (↑K_ATP_ channel)	PMV neuron (↑TRPC channel)
Insulin	POMC neuron (↑K_ATP_ channel)	NPY/AgRP neuron (N.D.)
	NPY/AgRP neuron (↑K_ATP_ channel)	
	SF1 neuron (↑K_ATP_ channel)	
Serotonin	NPY/AgRP neuron (via 5-HT_1B_Rs; N.D.)	POMC neuron (via 5-HT_2C_Rs; ↑TRPC channel, ↓M channel)
Ghrelin	^**^N.A.	NPY/AgRP neuron (N.D.)
Melanocortin	DMV neuron (via MC4Rs; ↑K_ATP_ channel)	PVH/DMH neuron (via MC3R/4Rs; N.D.)
		POMC neuron (via MC3R/4Rs; ↓multiple K^+^ channels)
		IML neuron (via MC4Rs; N.D.)
NPY	POMC neuron (↑GIRK channel)	N.A.
	Arcuate nucleus GABAergic neuron (↑GIRK channel)	
	VMH glutamatergic neuron (↑GIRK channel)	
	Orexin/Hypocretin neuron (↑GIRK channel)	

* N.D., involved ion channels not determined;

** N.A., data not available.

By contrast to the glucose-excitation of LHA MCH neurons (Burdakov et al., 2005; Kong et al., 2010), orexin/hypocretin neurons in LHA are inhibited by elevated glucose concentrations (glucose-inhibited or glucose-sensitive neurons) (Burdakov et al., 2005, 2006). The identity of ion channels underlying glucose-inhibition is still in debate. Earlier studies suggested the involvement of Na^+^-K^+^ ATPase and cystic fibrosis transmembrane regulator (CFTR)-like chloride conductance in the glucose inhibition (Oomura et al., 1974; Song et al., 2001; Routh, 2002; Fioramonti et al., 2007) (Table 1). Later, two-pore or tandem-pore domain K^+^ (K2P) channels were demonstrated to inhibit neuronal activity by elevated glucose concentrations in the orexin/hypocretin neurons of LHA (Burdakov et al., 2006) (Table 1). They claimed that TASK3 subunits, a subfamily of K2P channels, are responsible for the observed glucose-inhibition (Burdakov et al., 2006). However, genetic deletions of TASK1/TASK3 or TREK1/TREK2/TRAAK did not prevent glucose inhibition of orexin/hypocretin neurons (Guyon et al., 2009). These conflicting results highlight the requirement of better pharmacological and/or genetic tools to identify the molecular entity of ion channels underlying glucose inhibition of hypothalamic neurons.

***K_ATP_ channels mediate the acute inhibitory effects of leptin and insulin***. K_ATP_ channels also mediate metabolic effects of leptin and insulin, the anorexigenic hormones released from adipocytes and pancreatic beta cells, respectively (Williams et al., 2011a). In the arcuate nucleus, enhanced PIP_3_ signaling and increased K_ATP_ channel activity in POMC neurons led to hyperphagia and diet-induced obesity at least in part by blunting the acute effects of leptin and insulin (Plum et al., 2006). In the VMH, insulin receptors expressed by the SF-1 neurons were shown to activate K_ATP_ channels and suppress SF-1 neuron activity, which resulted in diet-induced obesity (Klockener et al., 2011). More recently, increased mTOR signaling was associated with elevated K_ATP_ channel activity in arcuate POMC neurons resulting in the cellular inhibition of arcuate POMC neurons concomitant with age-dependent obesity (Yang et al., 2012).

**Figure 2:**
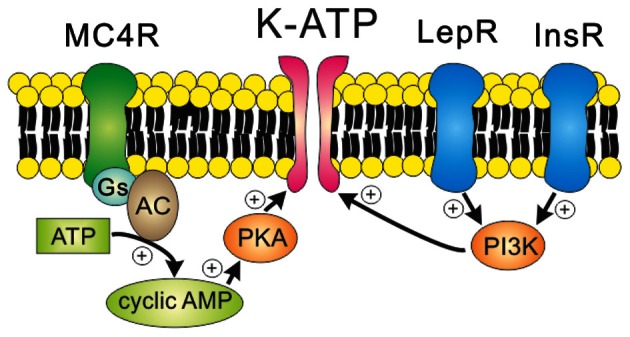
**Receptor-mediated regulation of K_ATP_ channels.** MC4Rs, which are Gs protein-coupled receptors, activate K_ATP_ channels via the cAMP/PKA signaling pathways. On the other hand, leptin receptors (type 1 cytokine receptors) and insulin receptors (class 2 tyrosine kinase receptors) activate K_ATP_ channels via the PI3K signaling pathway.

Leptin is well known for its anti-obesity and anti-diabetic effects (Zhang et al., 1994; Campfield et al., 1995; Farooqi et al., 1999). Evidence suggests that leptin effects are largely mediated via mechanisms in the CNS (Campfield et al., 1995; Halaas et al., 1995, 1997; Cohen et al., 2001; Spiegelman and Flier, 2001). Similarly, insulin levels in the brain are increased in proportion to blood levels, and some of insulin effects are mediated via mechanisms in the CNS (Woods et al., 1979; Obici et al., 2002). While leptin and insulin effects on energy and glucose homeostasis are largely mediated by Jak2/STAT3 and Akt/FOXO1 signaling cascades, respectively (Belgardt and Bruning, 2010), they also require phosphatidylinositol-3-kinase (PI3K) activity (Niswender et al., 2001; Zhao et al., 2002; Rahmouni et al., 2003; Mirshamsi et al., 2004; Morrison et al., 2005; Morton et al., 2005; Fukuda et al., 2008). Interestingly, the acute cellular effects of leptin and insulin are also mediated via PI3K activation (Choudhury et al., 2005; Plum et al., 2006; Hill et al., 2008; Al-Qassab et al., 2009; Klockener et al., 2011; Williams et al., 2011b) (Figure 2). The acute inhibition of cellular activity by leptin is mediated by the activation of K_ATP_ channels, as demonstrated by findings in several types of neurons located in hypothalamus and brainstem (Spanswick et al., 1997; Williams and Smith, 2006; Cui et al., 2012) (Figure 2 and Table 1). Typically, arcuate POMC neurons and leptin receptor (LepR)-expressing neurons of ventral premammilary nucleus (PMV) are activated by leptin (Cowley et al., 2001; Al-Qassab et al., 2009; Williams et al., 2010, 2011b) (Table 1). However, a minor population of arcuate POMC neurons and PMV LepR neurons is inhibited by leptin via K_ATP_ channel activation (Williams et al., 2010, 2011b) (Table 1). By contrast to leptin effects, acute effects of insulin are mostly inhibitory and mediated via the activation of K_ATP_ channels in neurons of VMH and arcuate nucleus (Spanswick et al., 2000; Hill et al., 2008; Williams et al., 2010; Klockener et al., 2011) (Figure 2 and Table 1). It was also reported, however, that insulin activated some arcuate neuropeptide Y (NPY)/agouti-related peptide (AgRP) neurons (Al-Qassab et al., 2009) (Table 1). In addition, although PI3K closely interact with Jak2/STAT3 and Akt/FOXO1 signaling cascades (Belgardt and Bruning, 2010; Williams et al., 2011a), there is currently no available data liking these signaling cascades to PI3K/K_ATP_ channel activation. Thus, while PI3K mediates the acute effects of leptin and insulin, more efforts are required to better understand the ion channel mechanisms and relevant signal pathways.

Arcuate POMC and NPY/AgRP neurons and VMH SF1 neurons are common targets of leptin and insulin (Al-Qassab et al., 2009; Williams et al., 2010; Klockener et al., 2011). As discussed, the acute effects of both leptin and insulin are mediated by the PI3K-dependent signaling pathways (Al-Qassab et al., 2009; Klockener et al., 2011; Williams et al., 2011b). However, their cellular effects are heterogeneous depending on their target neurons. POMC neurons and SF1 neurons are either activated or inhibited by leptin, and inhibited by insulin (Al-Qassab et al., 2009; Williams et al., 2010; Klockener et al., 2011). NPY/AgRP neurons are inhibited by leptin, but either inhibited or activated by insulin (Al-Qassab et al., 2009; Williams et al., 2010). Interestingly, leptin-activated, leptin-inhibited, and insulin-inhibited neurons have been found to be distinct within the POMC neuron population (Williams et al., 2010) and SF1 neuron population (Klockener et al., 2011). Thus, while leptin and insulin share the PI3K signaling pathways for their acute effects, it looks like they act on distinct populations of neurons. Given the divergent axonal projections of these neurons (Canteras et al., 1994; Baker and Herkenham, 1995; Elias et al., 1998, 2001; Bagnol et al., 1999), it will be an interesting focus of future investigation to delineate the relationship between leptin/insulin responsiveness of these neurons and their axonal projections.

***K_ATP_ channels mediate the acute inhibitory effects of melanocotins***. Melanocortin 4 receptors (MC4Rs) are important regulators of energy and glucose homeostasis (Huszar et al., 1997; Vaisse et al., 1998; Yeo et al., 1998; Farooqi et al., 2003). MC4Rs are expressed by distinct nuclei in the CNS (Kishi et al., 2003; Liu et al., 2003). Interestingly, MC4Rs in the paraventricular hypothalamic nucleus (PVH) decrease food intake whereas MC4Rs expressed by cholinergic neurons (including parasympathetic and sympathetic preganglionic neurons) increase energy expenditure and regulate glucose homeostasis, suggesting a divergence of the central melanocortin pathways (Balthasar et al., 2005; Rossi et al., 2011). MC4Rs are demonstrated to depolarize neurons within the PVH and dorsomedial hypothalamic nucleus (DMH) (Liu et al., 2003), but little is known about the specific ion channel mediating the acute effects of MC4Rs in these neurons (Table 1). It was suggested that the inhibition of multiple potassium channels underlies MC4R-induced depolarization of arcuate POMC neurons (Smith et al., 2007) (Table 1). In POMC neurons, however, a distinct physiological role of MC4Rs has not yet been characterized. A recent study demonstrated that MC4Rs hyperpolarize the parasympathetic preganglionic neurons in brainstem via PKA-dependent activation of K_ATP_ channels (Sohn et al., 2013) (Figure 2 and Table 1). Interestingly, it was shown in the same study that MC4Rs depolarize the sympathetic preganglionic neurons in spinal cord (Sohn et al., 2013) (Table 1). These results represent a reciprocal regulation of autonomic preganglionic neurons. The identity of ion channels underlying the depolarization of PVH/DMH neurons and the sympathetic preganglionic neurons has not yet been determined. Further experiments will be necessary to delineate the intracellular signal pathways and the target ion channels underlying the cellular effects of MC4Rs in CNS.

#### G protein-gated inwardly rectifying K^+^ (GIRK) channels

GIRK (Kir3) channels are a subfamily of inwardly rectifying K^+^ channels gated by G protein-coupled receptors (Hibino et al., 2010). GIRK channels are important regulators of RMP and cellular excitability in the heart and brain (Luscher et al., 1997; Wickman et al., 1998; Cruz et al., 2004; Luscher and Slesinger, 2010). There are four mammalian GIRK channel subunits, GIRK1~GIRK4. GIRK1/GIRK2 heterotetramers serve as the neuronal GIRK channel prototype, while cardiac GIRK channels are GIRK1/GIRK4 heterotetramers (Koyrakh et al., 2005; Luscher and Slesinger, 2010). Since GIRK1 homomers fail to form a functional GIRK channel, the knockout of GIRK2 subunits eliminates most GIRK currents in brain (Krapivinsky et al., 1995; Hedin et al., 1996; Kennedy et al., 1996, 1999; Ma et al., 2002). However, deficiency in GIRK2 subunits did not affect RMP of arcuate POMC neurons (Sohn et al., 2011). Notably, GIRK1 subunits were found to be largely responsible for stabilizing the membrane potential of arcuate POMC neurons (Sohn et al., 2011). In addition, global deficiency of GIRK4 subunits resulted in mice that developed late onset obesity through hypothalamic mechanisms (Perry et al., 2008). Thus, subunit composition of hypothalamic GIRK channels may be distinct from those identified in other brain areas including hippocampus and midbrain.

**Figure 3:**
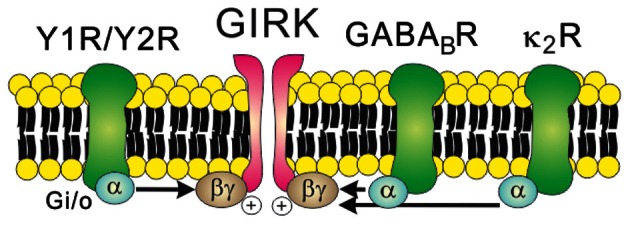
**Receptor-mediated regulation of GIRK channels.** GIRK channels are activated by the direct binding of Gβγ subunits in a membrane-delimited manner. Typically, Gβγ subunits which open GIRK channels originate from the Gi/o protein-coupled receptors such as Y receptors, GABA_B_ receptors, and κ_2_ opioid receptors.

GIRK channels may be constitutively active (Chen and Johnston, 2005; Sohn et al., 2011), but they are more commonly activated by the direct binding of Gβγ subunits originating from G_*i/o*_ protein-coupled receptors including Y receptors, GABA_B_ receptors, and opioid receptors (Figure 3). For instance, the hyperpolarizing effects of Y1R/Y2R are mediated by the activation of GIRK channels in the orexin/hypocretin neurons within LHA (Fu et al., 2004), the glutamatergic neurons within VMH (Chee et al., 2010) and the GABAergic neurons and POMC neurons within arcuate nucleus (Roseberry et al., 2004; Acuna-Goycolea et al., 2005) (Table 1). Recent evidence demonstrated that dynorphin, an endogenous opioid neuropeptide, inhibits arcuate POMC neurons via activation of κ_2_ opioid receptors and GIRK channels (Zhang and Van Den Pol, 2013). Likewise, GIRK channels are likely to underlie the slow inhibitory effects of serotonin 1B receptors (5-HT_1B_Rs) on arcuate NPY/AgRP neurons (Heisler et al., 2006). GIRK channels also mediate slow inhibitory effects of the metabotropic GABA_B_ receptors in arcuate POMC neurons (Sohn et al., 2011). Recently it was shown that arcuate POMC neuronal activity was regulated by changes in the GABAergic inhibitory postsynaptic currents (IPSCs), which are chloride currents through the ionotropic GABA_A_ receptors. (Tong et al., 2008; Vong et al., 2011). It is also possible that changes in GABAergic neurotransmission may also regulate arcuate POMC neurons via the GABA_B_ receptors and GIRK channels. However, this possibility has not yet been tested. Identifying the role of specific GIRK channel subunits in hypothalamus will be an interesting focus of future investigation.

### Non-selective cation (NSC) channels

NSC channels refer to a collection of ion channels that permeate cations (e.g., Na^+^, K^+^, and Ca^2+^) without ion selectivity. The relative conductance to each cation is different for each channel, but they typically have a reversal potential around −20 mV (Cowley et al., 2001; Hill et al., 2008; Sohn et al., 2011). The transient receptor potential (TRP) channel is the largest and probably the best-studied NSC channel family (Wu et al., 2010). Mammalian homologues of *Drosophila* TRP channels have been classified into subfamilies including canonical (TRPC), vanilloid (TRPV), melastatin (TRPM), and others (Wu et al., 2010). TRP channels are expressed widely in the CNS (Wu et al., 2010), and specific functions for each channel are being discovered. Mice with defective TRPC3 channels showed cerebellar locomotive dysfunction (Hartmann et al., 2008). In addition, TRPC5 channel knockout mice showed decreased fear (Riccio et al., 2009), and TRPV1 channel was found to be responsible for addiction and pain (Grueter et al., 2010; Kim et al., 2012).

**Figure 4:**
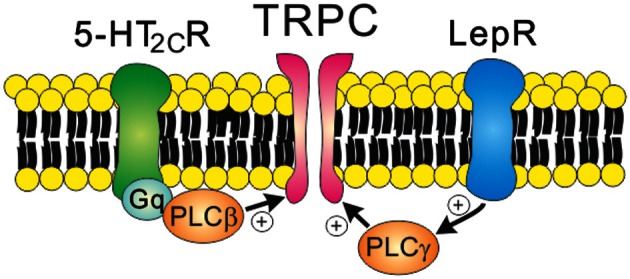
**Receptor-mediated regulation of TRPC channels.** 5-HT_2C_ receptors (Gq protein-coupled receptors) and leptin receptors (type 1 cytokine receptors) open TRPC channels via the PLC signaling pathway. Note that leptin receptors activate PI3K prior to PLC activation.

As discussed, leptin-induced hyperpolarization is attributed to the activation of K_ATP_ channels. By contrast, leptin depolarizes arcuate POMC neurons via the activation of NSC conductance (Cowley et al., 2001; Hill et al., 2008). It was later demonstrated that leptin-induced inward currents in POMC neurons were mediated by PI3K/PLC-dependent activation of TRPC channels (Qiu et al., 2010) (Figure 4 and Table 1). Depolarization of PMV LepR neurons and arcuate kiss1 neurons was also attributed to the activation of TRPC channels (Qiu et al., 2011; Williams et al., 2011b) (Table 1). Analyses of biophysical characteristics and single cell RT-PCR results suggested that TRPC4 and TRPC5 subunits, and TPRC6 to a lesser extent, may underlie leptin-induced neuronal activation (Qiu et al., 2010, 2011). Thus, leptin activation of central neurons is expected to be largely mediated via TRPC channels. However, the specific TRPC subunit mediating the acute effects of leptin (*in vitro* and *in vivo*) still remains undefined.

Serotonin 2C receptors (5-HT_2C_Rs) expressed by arcuate POMC neurons are important in mediating the anti-obesity and anti-diabetic effects of serotonin (Heisler et al., 2002; Xu et al., 2008, 2010). It was also suggested that 5-HT_1B_Rs expressed by arcuate NPY/AgRP neurons may underlie these effects of serotonin (Heisler et al., 2006). It was initially demonstrated that 5-HT_2C_Rs directly activate the anorexigenic POMC neurons (Heisler et al., 2002). Notably, 5-HT_1B_Rs directly inhibit the orexigenic NPY/AgRP neurons, which results in decreased IPSCs onto POMC neurons (Heisler et al., 2006). Thus, there is a local circuit involving arcuate POMC and NPY/AgRP neurons that mediates the anorexigenic effects of serotonin. More recently, it was shown that 5-HT_2C_Rs activate arcuate POMC neurons via PLC-dependent activation of TRPC channels (Sohn et al., 2011) (Figure 4). Thus, TRPC channels may serve as a common cellular target to mediate the acute effects of both leptin and serotonin (Figure 4 and Table 1). As suggested for acute effects of leptin and insulin (Williams et al., 2010), leptin and serotonin activated distinct subpopulations of arcuate POMC neurons (Sohn et al., 2011). The heterogeneity of arcuate POMC neurons may be related to the divergence of central melanocortin pathways (Balthasar et al., 2005; Sohn and Williams, 2012). It should be noted that 5-HT_2C_Rs also inhibit GIRK channels and M-type K^+^ channels in arcuate POMC neurons (Qiu et al., 2007; Roepke et al., 2012). Since these K^+^ channels stabilize neuronal RMP, the inhibition of these channels may also contribute to neuronal activation by 5-HT_2C_Rs (Delmas and Brown, 2005; Luscher and Slesinger, 2010).

In addition to leptin and serotonin, depolarization of arcuate NPY/AgRP neurons by neuromedin B and gastrin-releasing peptide is mediated by the activation of NSC conductance (Van Den Pol et al., 2009). Also, there are examples of membrane depolarization with unidentified ion channel mechanisms. For instance, ghrelin, an orexigenic hormone released from gastric mucosa, depolarizes arcuate NPY/AgRP neurons (Cowley et al., 2003) (Table 1). Moreover, MC4Rs are known to depolarize arcuate POMC neurons (Smith et al., 2007), neurons within the PVH and DMH (Liu et al., 2003), and the sympathetic preganglionic neurons (Sohn et al., 2013). Currently, it's unclear if these acute effects involve NSC channels such as TRP channels and cyclic nucleotide gated (CNG) channels.

### Ionotropic glutamate receptors (iGluRs) and GABA_A_ receptors

Neuronal excitability is frequently modulated by the excitatory neurotransmitter glutamate and the inhibitory neurotransmitter GABA released from presynaptic terminals (Pinto et al., 2004). Patch clamp electrophysiological recordings demonstrated abundant excitatory and inhibitory synaptic inputs onto both arcuate POMC neurons and NPY/AgRP neurons (Pinto et al., 2004; Sternson et al., 2005; Tong et al., 2008; Vong et al., 2011; Liu et al., 2012). The physiological importance of presynaptic glutamate release and postsynaptic iGluRs has been demonstrated in several studies. For instance, glutamate release machinery (vesicular glutamate transporter: vGluT2) in VMH SF-1 neurons was required to prevent hypoglycemia (Tong et al., 2007). In another study, food deprivation and ghrelin potentiated presynaptic glutamate release and increased spontaneous excitatory postsynaptic currents (sEPSCs) onto arcuate NPY/AgRP neurons (Yang et al., 2011). Recent studies highlighted the importance of NMDA receptors in fasting activation of arcuate NPY/AgRP neurons (Liu et al., 2012), and in relaying satiety signals from the nucleus tractus solitaries (NTS) to the parabrachial nucleus (PBN) (Wu et al., 2012).

Arcuate POMC neurons receive GABAergic input from multiple types of neurons including arcuate NPY/AgRP neurons (Cowley et al., 2001). The spontaneous IPSCs (sIPSCs) recorded in arcuate POMC neurons represented suppression of arcuate POMC neurons and alpha-MSH release (Tong et al., 2008; Vong et al., 2011). Defective synaptic GABA release from arcuate NPY/AgRP neurons produced a lean phenotype, which was associated with decreased IPSCs onto arcuate POMC neurons (Tong et al., 2008). In addition, deletion of LepRs in GABAergic neurons (probably non-NPY/AgRP) produced robust obesity (Vong et al., 2011), and this was associated with increased IPSCs onto arcuate POMC neurons. Notably, ghrelin increases IPSC frequency onto arcuate POMC neurons (Cowley et al., 2003; Tong et al., 2008), which may explain its orexigenic effects at least in part. Based on these results, it was suggested that GABA_A_ receptors expressed by arcuate POMC neurons may be important regulators of energy and glucose homeostasis. However, it should be noted that deletion of LepRs in POMC neurons, which may decrease neuronal firing frequency, resulted in only a mild obesity with no increase in food intake (Balthasar et al., 2004). In addition, recent studies demonstrated that GABAergic neurotransmission in the PVH and the PBN is important for the orexigenic effects of NPY/AgRP neuron stimulation (Wu et al., 2009; Atasoy et al., 2012). Thus, the observed alteration of IPSC frequency onto the arcuate POMC neurons may not be so critical as previously suggested for the metabolic effects observed in those mouse models (Cowley et al., 2003; Tong et al., 2008; Vong et al., 2011). Future studies may need to delineate the relative contribution of GABAergic neurotransmission and the role of GABA_A_ receptors expressed by arcuate POMC neurons and the neurons within PVH and PBN.

## Concluding remarks

In summary, multiple ion channels expressed by a specific neuron contribute to determine cellular response to humoral or synaptic inputs. For instance, K_ATP_ channels expressed by arcuate POMC neurons underlie the acute cellular inhibition by insulin receptors (Hill et al., 2008; Williams et al., 2010). On the other hand, the acute cellular activation by LepRs and 5-HT_2C_Rs are mediated by TRPC channels (Hill et al., 2008; Qiu et al., 2010; Williams et al., 2010; Sohn et al., 2011). In addition, GABA_A_ receptors are responsible for the fast inhibitory inputs (IPSCs) from NPY/AgRP and other GABAergic neurons (Cowley et al., 2001; Vong et al., 2011). Notably, these GABAergic neurons are modulated by leptin and ghrelin and therefore the changes in IPSCs recorded on arcuate POMC neurons may represent indirect effects of these hormones (Cowley et al., 2003; Pinto et al., 2004; Tong et al., 2008; Vong et al., 2011). Although direct evidence is lacking, it is possible that EPSCs recorded on arcuate POMC neurons may represent the glutamatergic input from VMH SF1 neurons (Sternson et al., 2005). Considering that glutamate release from SF1 neurons regulate glucose homeostasis (Tong et al., 2007), it will be worthwhile to study the role of iGluRs expressed by arcuate POMC neurons in glucose homeostasis. More specific functions of ion channels are expected to be discovered regarding the central regulation of food intake, energy expenditure, and glucose homeostasis.

Most of currently available data regarding the *in vivo* effects of hormones and neurotransmitters on metabolism was obtained by neuron-specific deletions of receptors or downstream signaling molecules using the Cre-loxP technology (Balthasar et al., 2005; Dhillon et al., 2006; Hill et al., 2008, 2010; Al-Qassab et al., 2009; Xu et al., 2010; Klockener et al., 2011; Rossi et al., 2011; Scott et al., 2011). While hormones and neurotransmitters frequently modulate ion channels *in vitro*, little is known about a specific function of ion channels in central regulation of metabolism *in vivo*. Recent evidence suggests that defective ion channel subunits in specific neuronal populations could lead to a long-term dysregulation of energy and glucose homeostasis (Parton et al., 2007; Liu et al., 2012). For a direct evaluation of how ion channels contribute to central regulation of metabolism, it will be reasonable to generate mice that have loxP-flanked ion channel genes and breed them with available Cre mouse models. Considering the importance of ion channels in neuronal and synaptic function, these studies will certainly advance our knowledge on the central mechanisms regulating energy and glucose homeostasis.

### Conflict of interest statement

The author declares that the research was conducted in the absence of any commercial or financial relationships that could be construed as a potential conflict of interest.
